# Supplementary material for: Immunomodulatory Properties of Immune Checkpoint Inhibitors—More than Boosting T-Cell Responses?
Source: Cancers (Basel). 2022 Mar 28;14(7):1710. doi: 10.3390/cancers14071710 (PMC8996886; doi:10.3390/cancers14071710)
Supplement: Supplementary file 1 [file cancers-14-01710-s001.zip › cancers-1626421-supplementary.pdf]

Supplementals

Immunomodulatory Properties of Immune Checkpoint Inhibitors—More than Boosting T-Cell Responses?

Michael Kuske †, Maximilian Haist †, Thomas Jung, Stephan Grabbe and Matthias Bros *

Department of Dermatology, University Medical Center Mainz, Langenbeckstraße 1, 55131 Mainz, Germany; mikuske@uni-mainz.de (M.K.); mhaist@uni-mainz.de (M.H.); thjung@students.uni-mainz.de (T.J.); stephan.grabbe@unimedizin-mainz.de (S.G.)

* Correspondence: mbros@uni-mainz.de; Tel.: +49-6131-17-9846

† These authors contributed equally to this work.

Supplementary Table S1. Expression pattern and immunological functions of further IC.

IC	Expressed by	Ligand	Expressed by	Outcome
CD96 [1]	T cells [2]<br>NK cells [3]<br>AML cells [4]	CD155 [3]	B cells [5]<br>DC [6]<br>follicular DC [5]<br>NK cells, NKT cells [5]<br>T cells [6]<br>tumor cells [7]<br>EC [8]	CTL activity ↓ [9]<br>CTL activity ↑ [10]<br>Th9 activity ↓ [11]<br>NK cell activity ↑ [3]<br>NK cell activity (IFN-γ) ↓ [12,13]
CD272 [14]	Th1 [15]<br>Tfh [16]<br>anergic T cells [17]<br>Th1, Th2, Th17 [18]<br>γδ T cells [19]<br>B cells [20]<br>activated DC [20]<br>macrophages [20]	HVEM [21]	B cells [22]<br>DC [22,23]<br>monocytes [24]<br>macrophages [25]<br>PMN [22,24]<br>T cells [26]<br>Treg [27]<br>EC [25]<br>fibroblasts [28]	T cell activity ↓ [20]<br>Th1 proliferation ↓[15]<br>T cell survival ↑ [29]<br>B cell activation ↓ [30]<br>macrophage, DC, PMN activity ↓ [31,32]
CD276 [33]	activated DC [34,35]<br>activated PMN [36]<br>airway EC [37]<br>fibroblasts [38]<br>muscle cells [39]<br>tumor cells [40]	TLT-2 [41,42]	B cells [43,44]<br>macrophages [44]<br>PMN [43]<br>Th, CD8+ T cells [41]	T cell activity ↓ [45]<br>T cell activity ↑ [34,46]<br>CD8+ T cell activity ↓ [47]<br>CD8+ T cell activity ↑ [41,42]<br>PMN activity ↑ [48]
CD278 [49]	Treg [50]<br>Tfh – Xu 2013<br>NKT cells [51]<br>ILC2 [52]<br>activated PMN [53]	CD275 [54]<br>CD28/CTLA-4 (human) [55]	DC [56-58]<br>(naive) B cells [59]<br>ILC-2 [52]<br>activated airway EC [60]<br>airway smooth muscle cells [61]<br>mesenchymal stem cells (MSC) [62]<br>osteoclasts [63]<br>tumor cells [64]	**CD278/CD275:**<br>T cell activity ↑ [56,65]<br>T cell activity (by EC) ↑ [66]<br>CTL activity ↑ [67]<br>T effector cells (IL-10) ↑ [57]<br>Th2 polarization ↑ [68,69]<br>Tfh differentiation ↑ [70]<br>Th17 (by intratumoral PMN) ↑ [53]

IC	Expressed by	Ligand	Expressed by	Outcome
				Treg expansion ↑ [71,72]<br>DC activity (Th17) ↑ [73,74]<br>B cell development and activity, GC formation,<br>Ig class switch ↓ [75-77]<br>Treg induction (by MSC) ↑ [62,78]<br>NKT survival/activity ↑ [51]<br>tumor cell migration ↓ [79]<br>ILC2 survival, activity ↑ [52]<br>smooth muscle cell expansion ↑ [61]<br>osteoclast differentiation/activity ↓ [63]<br>**CD278/CD28:**<br>T cell activity ↑ [55]
LAG-3 [80]	Th / CTL [81]<br>Treg [82]<br>B cells [83]<br>pDC[84]<br>NK cells [85]<br>activated NKT cells [86]	FGL1 [87,88]<br>Galectin-3 [89]<br>MHCII [90]	**MHCII:**<br>professional APC, inducible on other cell types [91]<br>**FGL1:**<br>hepatocytes, hepatocellular carcinoma [88,92]<br>**Galectin-3:**<br>numerous cell types [93]	**LAG-3 deficiency:**<br>DC activity ↑ [94]<br>NK cell activity ↓ [95]<br>NKT cell proliferation ↓ [86]<br>**LAG-3/Galectin-3:**<br>pDC expansion ↓ [84,89]<br>T cell proliferation ↓ [81,89,96]<br>**LAG-3/MHCII:**<br>DC activity ↑ [97,98]
TIGIT [99]	activated T cells,<br>Treg, memory T cells [100] NK cells [101]	CD112 [102]<br>CD155 [100]	**CD112:**<br>stimulated DC [103]<br>EC,<br>fibroblasts,<br>neurons [104]<br>tumor cells [105]<br>**CD115** ***(see above)***	**Fc-TIGIT/CD155:**<br>DC activity ↓ [100]<br>NK cell activity ↓ [101]<br>Treg activity ↑ [106]
TIM-3 [107]	CTL [108]	Ceacam-1 [109]<br>Galectin-9 [109]<br>HMGB1 [110]<br>PS [111]	**Ceacam-1:**<br>B cells, PMN, T cells [112]<br>EC [113,114]<br>tumor cells [115]	**TIM-3 ligand unknown:**<br>Treg activity ↑ [116]<br>DC and NK activity ↓ [117]<br>DC inflammasome ↓ [118]

IC	Expressed by	Ligand	Expressed by	Outcome
			**Galectin-9:**<br>Kupffer cells [119]<br>stimulated EC [120]<br>fibroblasts [121]<br>tumor cells [122]<br>**HMGB1:**<br>necrotic cells,<br>activated PMN [123]<br>**PS:**<br>apoptotic cells [124]	macrophages inflammasome ↓ [125]<br>**TIM-3/Ceacam-1:**<br>T cell exhaustion ↑ [126]<br>**TIM-3/Galectin-9:**<br>Th1 apoptosis ↑ [109]<br>DC activity ↑ [127]<br>NK activity ↑ [128]<br>NK activity ↓ [129]<br>DC and NKT cell expansion ↑ [130]<br>**TIM-3/HMGB1:**<br>DC activity ↓ [131]<br>**TIM-3/PS:**<br>T cell activity ↑ [132]
VISTA<br>[133]	Naive and memory CD4$^+$<br>T cells,<br>Treg,<br>monocytes and<br>macrophages,<br>DC,<br>PMN [134]<br>MDSC [135,136]<br>CD71$^+$ erythroid cells<br>[137,138]	Galectin-9 [139]<br>CD162 (PSGL-1)<br>(at acidic pH) [140]<br>VSIG3 [141]	**Galectin-9 (*see above*)**<br>**PSGL-1:**<br>T cells,<br>B cells [142]<br>PMN [143]<br>NK cells [144]<br>mast cells,<br>basophils [145]<br>osteoclasts [146]<br>tumor cells [147]<br>**VSIG3:**<br>neurons,<br>glia cells,<br>Sertoli cells,<br>germ cells [148,149]	**VISTA ligand unknown:**<br>CD4$^+$ T cell activity,<br>CD8$^+$ T cell activity ↑ [134]<br>Treg induction (by CD71$^+$ erythroid cells) ↑<br>[137,138]<br>(naive) T cell apoptosis ↑ [150] [139]<br>macrophages: chemokines ↓, chemokine receptors ↑<br>[151]<br>monocyte activity ↑ [152]<br>MDSC activity ↑ [136]

References

1. Jin, H.S.; Park, Y. Hitting the complexity of the TIGIT-CD96-CD112R-CD226 axis for next-generation cancer immunotherapy. *BMB Rep* **2021**, *54*, 2-11, doi:10.5483/BMBRep.2021.54.1.229.
2. Seth, S.; Maier, M.K.; Qiu, Q.; Ravens, I.; Kremmer, E.; Förster, R.; Bernhardt, G. The murine pan T cell marker CD96 is an adhesion receptor for CD155 and nectin-1. *Biochem Biophys Res Commun* **2007**, *364*, 959-965, doi:10.1016/j.bbrc.2007.10.102.
3. Fuchs, A.; Cella, M.; Giurisato, E.; Shaw, A.S.; Colonna, M. Cutting edge: CD96 (tactile) promotes NK cell-target cell adhesion by interacting with the poliovirus receptor (CD155). *J Immunol* **2004**, *172*, 3994-3998, doi:10.4049/jimmunol.172.7.3994.
4. Jiang, Y.; Xu, P.; Yao, D.; Chen, X.; Dai, H. CD33, CD96 and Death Associated Protein Kinase (DAPK) Expression Are Associated with the Survival Rate and/or Response to the Chemotherapy in the Patients with Acute Myeloid Leukemia (AML). *Med Sci Monit* **2017**, *23*, 1725-1732, doi:10.12659/msm.900305.
5. Lange, R.; Peng, X.; Wimmer, E.; Lipp, M.; Bernhardt, G. The poliovirus receptor CD155 mediates cell-to-matrix contacts by specifically binding to vitronectin. *Virology* **2001**, *285*, 218-227, doi:10.1006/viro.2001.0943.
6. Maier, M.K.; Seth, S.; Czeloth, N.; Qiu, Q.; Ravens, I.; Kremmer, E.; Ebel, M.; Müller, W.; Pabst, O.; Förster, R., et al. The adhesion receptor CD155 determines the magnitude of humoral immune responses against orally ingested antigens. *Eur J Immunol* **2007**, *37*, 2214-2225, doi:10.1002/eji.200737072.
7. O'Donnell, J.S.; Madore, J.; Li, X.Y.; Smyth, M.J. Tumor intrinsic and extrinsic immune functions of CD155. *Semin Cancer Biol* **2020**, *65*, 189-196, doi:10.1016/j.semcancer.2019.11.013.
8. Reymond, N.; Imbert, A.M.; Devilard, E.; Fabre, S.; Chabannon, C.; Xerri, L.; Farnarier, C.; Cantoni, C.; Bottino, C.; Moretta, A., et al. DNAM-1 and PVR regulate monocyte migration through endothelial junctions. *J Exp Med* **2004**, *199*, 1331-1341, doi:10.1084/jem.20032206.
9. Mittal, D.; Lepletier, A.; Madore, J.; Aguilera, A.R.; Stannard, K.; Blake, S.J.; Whitehall, V.L.J.; Liu, C.; Bettington, M.L.; Takeda, K., et al. CD96 Is an Immune Checkpoint That Regulates CD8(+) T-cell Antitumor Function. *Cancer Immunol Res* **2019**, *7*, 559-571, doi:10.1158/2326-6066.Cir-18-0637.
10. Chiang, E.Y.; de Almeida, P.E.; de Almeida Nagata, D.E.; Bowles, K.H.; Du, X.; Chitre, A.S.; Banta, K.L.; Kwon, Y.; McKenzie, B.; Mittman, S., et al. CD96 functions as a co-stimulatory receptor to enhance CD8(+) T cell activation and effector responses. *Eur J Immunol* **2020**, *50*, 891-902, doi:10.1002/eji.201948405.
11. Stanko, K.; Iwert, C.; Appelt, C.; Vogt, K.; Schumann, J.; Strunk, F.J.; Ahrlich, S.; Schlickeiser, S.; Romagnani, C.; Jürchott, K., et al. CD96 expression determines the inflammatory potential of IL-9-producing Th9 cells. *Proc Natl Acad Sci U S A* **2018**, *115*, E2940-e2949, doi:10.1073/pnas.1708329115.
12. Chan, C.J.; Martinet, L.; Gilfillan, S.; Souza-Fonseca-Guimaraes, F.; Chow, M.T.; Town, L.; Ritchie, D.S.; Colonna, M.; Andrews, D.M.; Smyth, M.J. The receptors CD96 and CD226 oppose each other in the regulation of natural killer cell functions. *Nat Immunol* **2014**, *15*, 431-438, doi:10.1038/ni.2850.

13. Blake, S.J.; Stannard, K.; Liu, J.; Allen, S.; Yong, M.C.; Mittal, D.; Aguilera, A.R.; Miles, J.J.; Lutzky, V.P.; de Andrade, L.F., et al. Suppression of Metastases Using a New Lymphocyte Checkpoint Target for Cancer Immunotherapy. *Cancer Discov* **2016**, *6*, 446-459, doi:10.1158/2159-8290.Cd-15-0944.
14. Demerlé, C.; Gorvel, L.; Olive, D. BTLA-HVEM Couple in Health and Diseases: Insights for Immunotherapy in Lung Cancer. *Front Oncol* **2021**, *11*, 682007, doi:10.3389/fonc.2021.682007.
15. Watanabe, N.; Gavrieli, M.; Sedy, J.R.; Yang, J.; Fallarino, F.; Loftin, S.K.; Hurchla, M.A.; Zimmerman, N.; Sim, J.; Zang, X., et al. BTLA is a lymphocyte inhibitory receptor with similarities to CTLA-4 and PD-1. *Nat Immunol* **2003**, *4*, 670-679, doi:10.1038/ni944.
16. M'Hidi, H.; Thibult, M.L.; Chetaille, B.; Rey, F.; Bouadallah, R.; Nicollas, R.; Olive, D.; Xerri, L. High expression of the inhibitory receptor BTLA in T-follicular helper cells and in B-cell small lymphocytic lymphoma/chronic lymphocytic leukemia. *Am J Clin Pathol* **2009**, *132*, 589-596, doi:10.1309/ajcpphkgyyggl39c.
17. Hurchla, M.A.; Sedy, J.R.; Gavrieli, M.; Drake, C.G.; Murphy, T.L.; Murphy, K.M. B and T lymphocyte attenuator exhibits structural and expression polymorphisms and is highly Induced in anergic CD4+ T cells. *J Immunol* **2005**, *174*, 3377-3385, doi:10.4049/jimmunol.174.6.3377.
18. Oster, C.; Wilde, B.; Specker, C.; Sun, M.; Kribben, A.; Witzke, O.; Dolff, S. BTLA Expression on Th1, Th2 and Th17 Effector T-Cells of Patients with Systemic Lupus Erythematosus Is Associated with Active Disease. *Int J Mol Sci* **2019**, *20*, doi:10.3390/ijms20184505.
19. Hwang, H.J.; Lee, J.J.; Kang, S.H.; Suh, J.K.; Choi, E.S.; Jang, S.; Hwang, S.H.; Koh, K.N.; Im, H.J.; Kim, N. The BTLA and PD-1 signaling pathways independently regulate the proliferation and cytotoxicity of human peripheral blood γδ T cells. *Immun Inflamm Dis* **2021**, *9*, 274-287, doi:10.1002/iid3.390.
20. Han, P.; Goularte, O.D.; Rufner, K.; Wilkinson, B.; Kaye, J. An inhibitory Ig superfamily protein expressed by lymphocytes and APCs is also an early marker of thymocyte positive selection. *J Immunol* **2004**, *172*, 5931-5939, doi:10.4049/jimmunol.172.10.5931.
21. Ning, Z.; Liu, K.; Xiong, H. Roles of BTLA in Immunity and Immune Disorders. *Front Immunol* **2021**, *12*, 654960, doi:10.3389/fimmu.2021.654960.
22. Jung, H.W.; La, S.J.; Kim, J.Y.; Heo, S.K.; Kim, J.Y.; Wang, S.; Kim, K.K.; Lee, K.M.; Cho, H.R.; Lee, H.W., et al. High levels of soluble herpes virus entry mediator in sera of patients with allergic and autoimmune diseases. *Exp Mol Med* **2003**, *35*, 501-508, doi:10.1038/emm.2003.65.
23. De Trez, C.; Schneider, K.; Potter, K.; Droin, N.; Fulton, J.; Norris, P.S.; Ha, S.W.; Fu, Y.X.; Murphy, T.; Murphy, K.M., et al. The inhibitory HVEM-BTLA pathway counter regulates lymphotoxin receptor signaling to achieve homeostasis of dendritic cells. *J Immunol* **2008**, *180*, 238-248, doi:10.4049/jimmunol.180.1.238.
24. Heo, S.K.; Ju, S.A.; Lee, S.C.; Park, S.M.; Choe, S.Y.; Kwon, B.; Kwon, B.S.; Kim, B.S. LIGHT enhances the bactericidal activity of human monocytes and neutrophils via HVEM. *J Leukoc Biol* **2006**, *79*, 330-338, doi:10.1189/jlb.1104694.
25. Xu, H.; Cao, D.; Guo, G.; Ruan, Z.; Wu, Y.; Chen, Y. The intrahepatic expression and distribution of BTLA and its ligand HVEM in patients with HBV-related acute-on-chronic liver failure. *Diagn Pathol* **2012**, *7*, 142, doi:10.1186/1746-1596-7-142.
26. Morel, Y.; Schiano de Colella, J.M.; Harrop, J.; Deen, K.C.; Holmes, S.D.; Wattam, T.A.; Khandekar, S.S.; Truneh, A.; Sweet, R.W.; Gastaut, J.A., et al. Reciprocal expression of the

TNF family receptor herpes virus entry mediator and its ligand LIGHT on activated T cells: LIGHT down-regulates its own receptor. *J Immunol* **2000**, *165*, 4397-4404, doi:10.4049/jimmunol.165.8.4397.
27. Tao, R.; Wang, L.; Murphy, K.M.; Fraser, C.C.; Hancock, W.W. Regulatory T cell expression of herpesvirus entry mediator suppresses the function of B and T lymphocyte attenuator-positive effector T cells. *J Immunol* **2008**, *180*, 6649-6655, doi:10.4049/jimmunol.180.10.6649.
28. Hosokawa, Y.; Hosokawa, I.; Ozaki, K.; Nakae, H.; Matsuo, T. TNFSF14 coordinately enhances CXCL10 and CXCL11 productions from IFN-gamma-stimulated human gingival fibroblasts. *Mol Immunol* **2010**, *47*, 666-670, doi:10.1016/j.molimm.2009.10.018.
29. Hurchla, M.A.; Sedy, J.R.; Murphy, K.M. Unexpected role of B and T lymphocyte attenuator in sustaining cell survival during chronic allostimulation. *J Immunol* **2007**, *178*, 6073-6082, doi:10.4049/jimmunol.178.10.6073.
30. Vendel, A.C.; Calemine-Fenaux, J.; Izrael-Tomasevic, A.; Chauhan, V.; Arnott, D.; Eaton, D.L. B and T lymphocyte attenuator regulates B cell receptor signaling by targeting Syk and BLNK. *J Immunol* **2009**, *182*, 1509-1517, doi:10.4049/jimmunol.182.3.1509.
31. Shubin, N.J.; Chung, C.S.; Heffernan, D.S.; Irwin, L.R.; Monaghan, S.F.; Ayala, A. BTLA expression contributes to septic morbidity and mortality by inducing innate inflammatory cell dysfunction. *J Leukoc Biol* **2012**, *92*, 593-603, doi:10.1189/jlb.1211641.
32. Cheng, T.; Bai, J.; Chung, C.S.; Chen, Y.; Biron, B.M.; Ayala, A. Enhanced Innate Inflammation Induced by Anti-BTLA Antibody in Dual Insult Model of Hemorrhagic Shock/Sepsis. *Shock* **2016**, *45*, 40-49, doi:10.1097/shk.0000000000000479.
33. Kontos, F.; Michelakos, T.; Kurokawa, T.; Sadagopan, A.; Schwab, J.H.; Ferrone, C.R.; Ferrone, S. B7-H3: An Attractive Target for Antibody-based Immunotherapy. *Clin Cancer Res* **2021**, *27*, 1227-1235, doi:10.1158/1078-0432.Ccr-20-2584.
34. Chapoval, A.I.; Ni, J.; Lau, J.S.; Wilcox, R.A.; Flies, D.B.; Liu, D.; Dong, H.; Sica, G.L.; Zhu, G.; Tamada, K., et al. B7-H3: a costimulatory molecule for T cell activation and IFN-gamma production. *Nat Immunol* **2001**, *2*, 269-274, doi:10.1038/85339.
35. Suh, W.K.; Gajewska, B.U.; Okada, H.; Gronski, M.A.; Bertram, E.M.; Dawicki, W.; Duncan, G.S.; Bukczynski, J.; Plyte, S.; Elia, A., et al. The B7 family member B7-H3 preferentially down-regulates T helper type 1-mediated immune responses. *Nat Immunol* **2003**, *4*, 899-906, doi:10.1038/ni967.
36. Li, Z.Y.; Wang, J.T.; Chen, G.; Shan, Z.G.; Wang, T.T.; Shen, Y.; Chen, J.; Yan, Z.B.; Peng, L.S.; Mao, F.Y., et al. Expression, regulation and clinical significance of B7-H3 on neutrophils in human gastric cancer. *Clin Immunol* **2021**, *227*, 108753, doi:10.1016/j.clim.2021.108753.
37. Kim, J.; Myers, A.C.; Chen, L.; Pardoll, D.M.; Truong-Tran, Q.A.; Lane, A.P.; McDyer, J.F.; Fortuno, L.; Schleimer, R.P. Constitutive and inducible expression of b7 family of ligands by human airway epithelial cells. *Am J Respir Cell Mol Biol* **2005**, *33*, 280-289, doi:10.1165/rcmb.2004-0129OC.
38. Tran, C.N.; Thacker, S.G.; Louie, D.M.; Oliver, J.; White, P.T.; Endres, J.L.; Urquhart, A.G.; Chung, K.C.; Fox, D.A. Interactions of T cells with fibroblast-like synoviocytes: role of the B7 family costimulatory ligand B7-H3. *J Immunol* **2008**, *180*, 2989-2998, doi:10.4049/jimmunol.180.5.2989.

39. Waschbisch, A.; Wintterle, S.; Lochmüller, H.; Walter, M.C.; Wischhusen, J.; Kieseier, B.C.; Wiendl, H. Human muscle cells express the costimulatory molecule B7-H3, which modulates muscle-immune interactions. *Arthritis Rheum* **2008**, *58*, 3600-3608, doi:10.1002/art.23997.
40. Chen, Y.W.; Tekle, C.; Fodstad, O. The immunoregulatory protein human B7H3 is a tumor-associated antigen that regulates tumor cell migration and invasion. *Curr Cancer Drug Targets* **2008**, *8*, 404-413, doi:10.2174/156800908785133141.
41. Hashiguchi, M.; Kobori, H.; Ritprajak, P.; Kamimura, Y.; Kozono, H.; Azuma, M. Triggering receptor expressed on myeloid cell-like transcript 2 (TLT-2) is a counter-receptor for B7-H3 and enhances T cell responses. *Proc Natl Acad Sci U S A* **2008**, *105*, 10495-10500, doi:10.1073/pnas.0802423105.
42. Kobori, H.; Hashiguchi, M.; Piao, J.; Kato, M.; Ritprajak, P.; Azuma, M. Enhancement of effector CD8+ T-cell function by tumour-associated B7-H3 and modulation of its counter-receptor triggering receptor expressed on myeloid cell-like transcript 2 at tumour sites. *Immunology* **2010**, *130*, 363-373, doi:10.1111/j.1365-2567.2009.03236.x.
43. King, R.G.; Herrin, B.R.; Justement, L.B. Trem-like transcript 2 is expressed on cells of the myeloid/granuloid and B lymphoid lineage and is up-regulated in response to inflammation. *J Immunol* **2006**, *176*, 6012-6021, doi:10.4049/jimmunol.176.10.6012.
44. Li, J.; Cao, C.; Xiang, Y.; Hong, Z.; He, D.; Zhong, H.; Liu, Y.; Wu, Y.; Zheng, X.; Yin, H., et al. TLT2 Suppresses Th1 Response by Promoting IL-6 Production in Monocyte Through JAK/STAT3 Signal Pathway in Tuberculosis. *Front Immunol* **2020**, *11*, 2031, doi:10.3389/fimmu.2020.02031.
45. Leitner, J.; Klauser, C.; Pickl, W.F.; Stöckl, J.; Majdic, O.; Bardet, A.F.; Kreil, D.P.; Dong, C.; Yamazaki, T.; Zlabinger, G., et al. B7-H3 is a potent inhibitor of human T-cell activation: No evidence for B7-H3 and TREML2 interaction. *Eur J Immunol* **2009**, *39*, 1754-1764, doi:10.1002/eji.200839028.
46. Yan, R.; Yang, S.; Gu, A.; Zhan, F.; He, C.; Qin, C.; Zhang, X.; Feng, P. Murine b7-h3 is a co-stimulatory molecule for T cell activation. *Monoclon Antib Immunodiagn Immunother* **2013**, *32*, 395-398, doi:10.1089/mab.2013.0052.
47. Xu, J.C.; Gao, F.; Liu, Y.A.; Zhang, X.L.; Chen, H.; Zhu, X.Y.; Song, H.F.; Qian, F.; Li, M.; Yang, C., et al. Myeloid cell-like transcript 2 is related to liver inflammation and the pathogenesis of hepatitis B via the involvement of CD8(+)T cell activation. *Clin Exp Med* **2019**, *19*, 93-104, doi:10.1007/s10238-018-0534-1.
48. Halpert, M.M.; Thomas, K.A.; King, R.G.; Justement, L.B. TLT2 potentiates neutrophil antibacterial activity and chemotaxis in response to G protein-coupled receptor-mediated signaling. *J Immunol* **2011**, *187*, 2346-2355, doi:10.4049/jimmunol.1100534.
49. Amatore, F.; Gorvel, L.; Olive, D. Role of Inducible Co-Stimulator (ICOS) in cancer immunotherapy. *Expert Opin Biol Ther* **2020**, *20*, 141-150, doi:10.1080/14712598.2020.1693540.
50. Li, D.Y.; Xiong, X.Z. ICOS(+) Tregs: A Functional Subset of Tregs in Immune Diseases. *Front Immunol* **2020**, *11*, 2104, doi:10.3389/fimmu.2020.02104.
51. Akbari, O.; Stock, P.; Meyer, E.H.; Freeman, G.J.; Sharpe, A.H.; Umetsu, D.T.; DeKruyff, R.H. ICOS/ICOSL interaction is required for CD4+ invariant NKT cell function and homeostatic survival. *J Immunol* **2008**, *180*, 5448-5456, doi:10.4049/jimmunol.180.8.5448.
52. Maazi, H.; Patel, N.; Sankaranarayanan, I.; Suzuki, Y.; Rigas, D.; Soroosh, P.; Freeman, G.J.; Sharpe, A.H.; Akbari, O. ICOS:ICOS-ligand interaction is required for type 2 innate lymphoid

cell function, homeostasis, and induction of airway hyperreactivity. *Immunity* **2015**, *42*, 538-551, doi:10.1016/j.immuni.2015.02.007.

53. Shan, Z.G.; Chen, J.; Liu, J.S.; Zhang, J.Y.; Wang, T.T.; Teng, Y.S.; Mao, F.Y.; Cheng, P.; Zou, Q.M.; Zhou, W.Y., et al. Activated neutrophils polarize protumorigenic interleukin-17A-producing T helper subsets through TNF-α-B7-H2-dependent pathway in human gastric cancer. *Clin Transl Med* **2021**, *11*, e484, doi:10.1002/ctm2.484.
54. Ling, V.; Wu, P.W.; Finnerty, H.F.; Bean, K.M.; Spaulding, V.; Fouser, L.A.; Leonard, J.P.; Hunter, S.E.; Zollner, R.; Thomas, J.L., et al. Cutting edge: identification of GL50, a novel B7-like protein that functionally binds to ICOS receptor. *J Immunol* **2000**, *164*, 1653-1657, doi:10.4049/jimmunol.164.4.1653.
55. Yao, S.; Zhu, Y.; Zhu, G.; Augustine, M.; Zheng, L.; Goode, D.J.; Broadwater, M.; Ruff, W.; Flies, S.; Xu, H., et al. B7-h2 is a costimulatory ligand for CD28 in human. *Immunity* **2011**, *34*, 729-740, doi:10.1016/j.immuni.2011.03.014.
56. Wang, S.; Zhu, G.; Chapoval, A.I.; Dong, H.; Tamada, K.; Ni, J.; Chen, L. Costimulation of T cells by B7-H2, a B7-like molecule that binds ICOS. *Blood* **2000**, *96*, 2808-2813.
57. Witsch, E.J.; Peiser, M.; Hutloff, A.; Büchner, K.; Dorner, B.G.; Jonuleit, H.; Mages, H.W.; Kroczek, R.A. ICOS and CD28 reversely regulate IL-10 on re-activation of human effector T cells with mature dendritic cells. *Eur J Immunol* **2002**, *32*, 2680-2686, doi:10.1002/1521-4141(200209)32:9<2680::Aid-immu2680>3.0.Co;2-6.
58. Faget, J.; Bendriss-Vermare, N.; Gobert, M.; Durand, I.; Olive, D.; Biota, C.; Bachelot, T.; Treilleux, I.; Goddard-Leon, S.; Lavergne, E., et al. ICOS-ligand expression on plasmacytoid dendritic cells supports breast cancer progression by promoting the accumulation of immunosuppressive CD4+ T cells. *Cancer Res* **2012**, *72*, 6130-6141, doi:10.1158/0008-5472.Can-12-2409.
59. Liang, L.; Porter, E.M.; Sha, W.C. Constitutive expression of the B7h ligand for inducible costimulator on naive B cells is extinguished after activation by distinct B cell receptor and interleukin 4 receptor-mediated pathways and can be rescued by CD40 signaling. *J Exp Med* **2002**, *196*, 97-108, doi:10.1084/jem.20020298.
60. Kurosawa, S.; Myers, A.C.; Chen, L.; Wang, S.; Ni, J.; Plitt, J.R.; Heller, N.M.; Bochner, B.S.; Schleimer, R.P. Expression of the costimulatory molecule B7-H2 (inducible costimulator ligand) by human airway epithelial cells. *Am J Respir Cell Mol Biol* **2003**, *28*, 563-573, doi:10.1165/rcmb.2002-0199OC.
61. Kajiwara, K.; Morishima, H.; Akiyama, K.; Yanagihara, Y. Expression and function of the inducible costimulator ligand B7-H2 in human airway smooth muscle cells. *Allergol Int* **2009**, *58*, 573-583, doi:10.2332/allergolint.09-OA-0113.
62. Lee, H.J.; Kim, S.N.; Jeon, M.S.; Yi, T.; Song, S.U. ICOSL expression in human bone marrow-derived mesenchymal stem cells promotes induction of regulatory T cells. *Sci Rep* **2017**, *7*, 44486, doi:10.1038/srep44486.
63. Gigliotti, C.L.; Boggio, E.; Clemente, N.; Shivakumar, Y.; Toth, E.; Sblattero, D.; D'Amelio, P.; Isaia, G.C.; Dianzani, C.; Yagi, J., et al. ICOS-Ligand Triggering Impairs Osteoclast Differentiation and Function In Vitro and In Vivo. *J Immunol* **2016**, *197*, 3905-3916, doi:10.4049/jimmunol.1600424.

64. Martin-Orozco, N.; Li, Y.; Wang, Y.; Liu, S.; Hwu, P.; Liu, Y.J.; Dong, C.; Radvanyi, L. Melanoma cells express ICOS ligand to promote the activation and expansion of T-regulatory cells. *Cancer Res* **2010**, *70*, 9581-9590, doi:10.1158/0008-5472.Can-10-1379.
65. Takahashi, N.; Matsumoto, K.; Saito, H.; Nanki, T.; Miyasaka, N.; Kobata, T.; Azuma, M.; Lee, S.K.; Mizutani, S.; Morio, T. Impaired CD4 and CD8 effector function and decreased memory T cell populations in ICOS-deficient patients. *J Immunol* **2009**, *182*, 5515-5527, doi:10.4049/jimmunol.0803256.
66. Khayyamian, S.; Hutloff, A.; Büchner, K.; Gräfe, M.; Henn, V.; Kroczek, R.A.; Mages, H.W. ICOS-ligand, expressed on human endothelial cells, costimulates Th1 and Th2 cytokine secretion by memory CD4+ T cells. *Proc Natl Acad Sci U S A* **2002**, *99*, 6198-6203, doi:10.1073/pnas.092576699.
67. Wallin, J.J.; Liang, L.; Bakardjiev, A.; Sha, W.C. Enhancement of CD8+ T cell responses by ICOS/B7h costimulation. *J Immunol* **2001**, *167*, 132-139, doi:10.4049/jimmunol.167.1.132.
68. McAdam, A.J.; Chang, T.T.; Lumelsky, A.E.; Greenfield, E.A.; Boussiotis, V.A.; Duke-Cohan, J.S.; Chernova, T.; Malenkovich, N.; Jabs, C.; Kuchroo, V.K., et al. Mouse inducible costimulatory molecule (ICOS) expression is enhanced by CD28 costimulation and regulates differentiation of CD4+ T cells. *J Immunol* **2000**, *165*, 5035-5040, doi:10.4049/jimmunol.165.9.5035.
69. Nurieva, R.I.; Mai, X.M.; Forbush, K.; Bevan, M.J.; Dong, C. B7h is required for T cell activation, differentiation, and effector function. *Proc Natl Acad Sci U S A* **2003**, *100*, 14163-14168, doi:10.1073/pnas.2335041100.
70. Xu, H.; Li, X.; Liu, D.; Li, J.; Zhang, X.; Chen, X.; Hou, S.; Peng, L.; Xu, C.; Liu, W., et al. Follicular T-helper cell recruitment governed by bystander B cells and ICOS-driven motility. *Nature* **2013**, *496*, 523-527, doi:10.1038/nature12058.
71. Akbari, O.; Freeman, G.J.; Meyer, E.H.; Greenfield, E.A.; Chang, T.T.; Sharpe, A.H.; Berry, G.; DeKruyff, R.H.; Umetsu, D.T. Antigen-specific regulatory T cells develop via the ICOS-ICOS-ligand pathway and inhibit allergen-induced airway hyperreactivity. *Nat Med* **2002**, *8*, 1024-1032, doi:10.1038/nm745.
72. Han, Y.; Dong, Y.; Yang, Q.; Xu, W.; Jiang, S.; Yu, Z.; Yu, K.; Zhang, S. Acute Myeloid Leukemia Cells Express ICOS Ligand to Promote the Expansion of Regulatory T Cells. *Front Immunol* **2018**, *9*, 2227, doi:10.3389/fimmu.2018.02227.
73. Tang, G.; Qin, Q.; Zhang, P.; Wang, G.; Liu, M.; Ding, Q.; Qin, Y.; Shen, Q. Reverse signaling using an inducible costimulator to enhance immunogenic function of dendritic cells. *Cell Mol Life Sci* **2009**, *66*, 3067-3080, doi:10.1007/s00018-009-0090-7.
74. Occhipinti, S.; Dianzani, C.; Chiocchetti, A.; Boggio, E.; Clemente, N.; Gigliotti, C.L.; Soluri, M.F.; Minelli, R.; Fantozzi, R.; Yagi, J., et al. Triggering of B7h by the ICOS modulates maturation and migration of monocyte-derived dendritic cells. *J Immunol* **2013**, *190*, 1125-1134, doi:10.4049/jimmunol.1201816.
75. Dong, C.; Temann, U.A.; Flavell, R.A. Cutting edge: critical role of inducible costimulator in germinal center reactions. *J Immunol* **2001**, *166*, 3659-3662, doi:10.4049/jimmunol.166.6.3659.
76. Grimbacher, B.; Hutloff, A.; Schlesier, M.; Glocker, E.; Warnatz, K.; Dräger, R.; Eibel, H.; Fischer, B.; Schäffer, A.A.; Mages, H.W., et al. Homozygous loss of ICOS is associated with adult-onset common variable immunodeficiency. *Nat Immunol* **2003**, *4*, 261-268, doi:10.1038/ni902.

77. Warnatz, K.; Bossaller, L.; Salzer, U.; Skrabl-Baumgartner, A.; Schwinger, W.; van der Burg, M.; van Dongen, J.J.; Orlowska-Volk, M.; Knoth, R.; Durandy, A., et al. Human ICOS deficiency abrogates the germinal center reaction and provides a monogenic model for common variable immunodeficiency. *Blood* **2006**, *107*, 3045-3052, doi:10.1182/blood-2005-07-2955.
78. Carrión, F.; Nova, E.; Luz, P.; Apablaza, F.; Figueroa, F. Opposing effect of mesenchymal stem cells on Th1 and Th17 cell polarization according to the state of CD4+ T cell activation. *Immunol Lett* **2011**, *135*, 10-16, doi:10.1016/j.imlet.2010.09.006.
79. Dianzani, C.; Minelli, R.; Gigliotti, C.L.; Occhipinti, S.; Giovarelli, M.; Conti, L.; Boggio, E.; Shivakumar, Y.; Baldanzi, G.; Malacarne, V., et al. B7h triggering inhibits the migration of tumor cell lines. *J Immunol* **2014**, *192*, 4921-4931, doi:10.4049/jimmunol.1300587.
80. Lecocq, Q.; Keyaerts, M.; Devoogdt, N.; Breckpot, K. The Next-Generation Immune Checkpoint LAG-3 and Its Therapeutic Potential in Oncology: Third Time's a Charm. *Int J Mol Sci* **2020**, *22*, doi:10.3390/ijms22010075.
81. Workman, C.J.; Vignali, D.A. The CD4-related molecule, LAG-3 (CD223), regulates the expansion of activated T cells. *Eur J Immunol* **2003**, *33*, 970-979, doi:10.1002/eji.200323382.
82. Gagliani, N.; Magnani, C.F.; Huber, S.; Gianolini, M.E.; Pala, M.; Licona-Limon, P.; Guo, B.; Herbert, D.R.; Bulfone, A.; Trentini, F., et al. Coexpression of CD49b and LAG-3 identifies human and mouse T regulatory type 1 cells. *Nat Med* **2013**, *19*, 739-746, doi:10.1038/nm.3179.
83. Kisielow, M.; Kisielow, J.; Capoferri-Sollami, G.; Karjalainen, K. Expression of lymphocyte activation gene 3 (LAG-3) on B cells is induced by T cells. *Eur J Immunol* **2005**, *35*, 2081-2088, doi:10.1002/eji.200526090.
84. Workman, C.J.; Wang, Y.; El Kasmi, K.C.; Pardoll, D.M.; Murray, P.J.; Drake, C.G.; Vignali, D.A. LAG-3 regulates plasmacytoid dendritic cell homeostasis. *J Immunol* **2009**, *182*, 1885-1891, doi:10.4049/jimmunol.0800185.
85. Triebel, F.; Jitsukawa, S.; Baixeras, E.; Roman-Roman, S.; Genevee, C.; Viegas-Pequignot, E.; Hercend, T. LAG-3, a novel lymphocyte activation gene closely related to CD4. *J Exp Med* **1990**, *171*, 1393-1405, doi:10.1084/jem.171.5.1393.
86. Byun, H.J.; Jung, W.W.; Lee, D.S.; Kim, S.; Kim, S.J.; Park, C.G.; Chung, H.Y.; Chun, T. Proliferation of activated CD1d-restricted NKT cells is down-modulated by lymphocyte activation gene-3 signaling via cell cycle arrest in S phase. *Cell Biol Int* **2007**, *31*, 257-262, doi:10.1016/j.cellbi.2006.11.002.
87. Wang, J.; Sanmamed, M.F.; Datar, I.; Su, T.T.; Ji, L.; Sun, J.; Chen, L.; Chen, Y.; Zhu, G.; Yin, W., et al. Fibrinogen-like Protein 1 Is a Major Immune Inhibitory Ligand of LAG-3. *Cell* **2019**, *176*, 334-347.e312, doi:10.1016/j.cell.2018.11.010.
88. Qian, W.; Zhao, M.; Wang, R.; Li, H. Fibrinogen-like protein 1 (FGL1): the next immune checkpoint target. *J Hematol Oncol* **2021**, *14*, 147, doi:10.1186/s13045-021-01161-8.
89. Kouo, T.; Huang, L.; Pucsek, A.B.; Cao, M.; Solt, S.; Armstrong, T.; Jaffee, E. Galectin-3 Shapes Antitumor Immune Responses by Suppressing CD8+ T Cells via LAG-3 and Inhibiting Expansion of Plasmacytoid Dendritic Cells. *Cancer Immunol Res* **2015**, *3*, 412-423, doi:10.1158/2326-6066.Cir-14-0150.
90. Huard, B.; Mastrangeli, R.; Prigent, P.; Bruniquel, D.; Donini, S.; El-Tayar, N.; Maigret, B.; Dréano, M.; Triebel, F. Characterization of the major histocompatibility complex class II

binding site on LAG-3 protein. *Proc Natl Acad Sci U S A* **1997**, *94*, 5744-5749, doi:10.1073/pnas.94.11.5744.

91. León Machado, J.A.; Steimle, V. The MHC Class II Transactivator CIITA: Not (Quite) the Odd-One-Out Anymore among NLR Proteins. *Int J Mol Sci* **2021**, *22*, doi:10.3390/ijms22031074.
92. Hara, H.; Yoshimura, H.; Uchida, S.; Toyoda, Y.; Aoki, M.; Sakai, Y.; Morimoto, S.; Shiokawa, K. Molecular cloning and functional expression analysis of a cDNA for human hepassocin, a liver-specific protein with hepatocyte mitogenic activity. *Biochim Biophys Acta* **2001**, *1520*, 45-53, doi:10.1016/s0167-4781(01)00249-4.
93. Capone, E.; Iacobelli, S.; Sala, G. Role of galectin 3 binding protein in cancer progression: a potential novel therapeutic target. *J Transl Med* **2021**, *19*, 405, doi:10.1186/s12967-021-03085-w.
94. Garcia Cruz, D.; Giri, R.R.; Gamiotea Turro, D.; Balsbaugh, J.L.; Adler, A.J.; Rodriguez, A. Lymphocyte Activation Gene-3 Regulates Dendritic Cell Metabolic Programing and T Cell Priming Function. *J Immunol* **2021**, *207*, 2374-2384, doi:10.4049/jimmunol.2001188.
95. Miyazaki, T.; Dierich, A.; Benoist, C.; Mathis, D. Independent modes of natural killing distinguished in mice lacking Lag3. *Science* **1996**, *272*, 405-408, doi:10.1126/science.272.5260.405.
96. Workman, C.J.; Cauley, L.S.; Kim, I.J.; Blackman, M.A.; Woodland, D.L.; Vignali, D.A. Lymphocyte activation gene-3 (CD223) regulates the size of the expanding T cell population following antigen activation in vivo. *J Immunol* **2004**, *172*, 5450-5455, doi:10.4049/jimmunol.172.9.5450.
97. Avice, M.N.; Sarfati, M.; Triebel, F.; Delespesse, G.; Demeure, C.E. Lymphocyte activation gene-3, a MHC class II ligand expressed on activated T cells, stimulates TNF-alpha and IL-12 production by monocytes and dendritic cells. *J Immunol* **1999**, *162*, 2748-2753.
98. Andreae, S.; Piras, F.; Burdin, N.; Triebel, F. Maturation and activation of dendritic cells induced by lymphocyte activation gene-3 (CD223). *J Immunol* **2002**, *168*, 3874-3880, doi:10.4049/jimmunol.168.8.3874.
99. Ge, Z.; Peppelenbosch, M.P.; Sprengers, D.; Kwekkeboom, J. TIGIT, the Next Step Towards Successful Combination Immune Checkpoint Therapy in Cancer. *Front Immunol* **2021**, *12*, 699895, doi:10.3389/fimmu.2021.699895.
100. Yu, X.; Harden, K.; Gonzalez, L.C.; Francesco, M.; Chiang, E.; Irving, B.; Tom, I.; Ivelja, S.; Refino, C.J.; Clark, H., et al. The surface protein TIGIT suppresses T cell activation by promoting the generation of mature immunoregulatory dendritic cells. *Nat Immunol* **2009**, *10*, 48-57, doi:10.1038/ni.1674.
101. Stanietsky, N.; Simic, H.; Arapovic, J.; Toporik, A.; Levy, O.; Novik, A.; Levine, Z.; Beiman, M.; Dassa, L.; Achdout, H., et al. The interaction of TIGIT with PVR and PVRL2 inhibits human NK cell cytotoxicity. *Proc Natl Acad Sci U S A* **2009**, *106*, 17858-17863, doi:10.1073/pnas.0903474106.
102. Bottino, C.; Castriconi, R.; Pende, D.; Rivera, P.; Nanni, M.; Carnemolla, B.; Cantoni, C.; Grassi, J.; Marcenaro, S.; Reymond, N., et al. Identification of PVR (CD155) and Nectin-2 (CD112) as cell surface ligands for the human DNAM-1 (CD226) activating molecule. *J Exp Med* **2003**, *198*, 557-567, doi:10.1084/jem.20030788.

103. Zhu, Y.; Paniccia, A.; Schulick, A.C.; Chen, W.; Koenig, M.R.; Byers, J.T.; Yao, S.; Bevers, S.; Edil, B.H. Identification of CD112R as a novel checkpoint for human T cells. *J Exp Med* **2016**, *213*, 167-176, doi:10.1084/jem.20150785.
104. Takai, Y.; Nakanishi, H. Nectin and afadin: novel organizers of intercellular junctions. *J Cell Sci* **2003**, *116*, 17-27, doi:10.1242/jcs.00167.
105. Zeng, T.; Cao, Y.; Jin, T.; Tian, Y.; Dai, C.; Xu, F. The CD112R/CD112 axis: a breakthrough in cancer immunotherapy. *J Exp Clin Cancer Res* **2021**, *40*, 285, doi:10.1186/s13046-021-02053-y.
106. Kurtulus, S.; Sakuishi, K.; Ngiow, S.F.; Joller, N.; Tan, D.J.; Teng, M.W.; Smyth, M.J.; Kuchroo, V.K.; Anderson, A.C. TIGIT predominantly regulates the immune response via regulatory T cells. *J Clin Invest* **2015**, *125*, 4053-4062, doi:10.1172/jci81187.
107. Zhao, L.; Cheng, S.; Fan, L.; Zhang, B.; Xu, S. TIM-3: An update on immunotherapy. *Int Immunopharmacol* **2021**, *99*, 107933, doi:10.1016/j.intimp.2021.107933.
108. Qiu, Y.; Chen, J.; Liao, H.; Zhang, Y.; Wang, H.; Li, S.; Luo, Y.; Fang, D.; Li, G.; Zhou, B., et al. Tim-3-expressing CD4+ and CD8+ T cells in human tuberculosis (TB) exhibit polarized effector memory phenotypes and stronger anti-TB effector functions. *PLoS Pathog* **2012**, *8*, e1002984, doi:10.1371/journal.ppat.1002984.
109. Zhu, C.; Anderson, A.C.; Schubart, A.; Xiong, H.; Imitola, J.; Khoury, S.J.; Zheng, X.X.; Strom, T.B.; Kuchroo, V.K. The Tim-3 ligand galectin-9 negatively regulates T helper type 1 immunity. *Nat Immunol* **2005**, *6*, 1245-1252, doi:10.1038/ni1271.
110. Tang, D.; Lotze, M.T. Tumor immunity times out: TIM-3 and HMGB1. *Nat Immunol* **2012**, *13*, 808-810, doi:10.1038/ni.2396.
111. Weber, J.K.; Zhou, R. Phosphatidylserine-Induced Conformational Modulation of Immune Cell Exhaustion-Associated Receptor TIM3. *Sci Rep* **2017**, *7*, 13579, doi:10.1038/s41598-017-14064-x.
112. Singer, B.B.; Scheffrahn, I.; Heymann, R.; Sigmundsson, K.; Kammerer, R.; Obrink, B. Carcinoembryonic antigen-related cell adhesion molecule 1 expression and signaling in human, mouse, and rat leukocytes: evidence for replacement of the short cytoplasmic domain isoform by glycosylphosphatidylinositol-linked proteins in human leukocytes. *J Immunol* **2002**, *168*, 5139-5146, doi:10.4049/jimmunol.168.10.5139.
113. Ergün, S.; Kilik, N.; Ziegeler, G.; Hansen, A.; Nollau, P.; Götze, J.; Wurmbach, J.H.; Horst, A.; Weil, J.; Fernando, M., et al. CEA-related cell adhesion molecule 1: a potent angiogenic factor and a major effector of vascular endothelial growth factor. *Mol Cell* **2000**, *5*, 311-320, doi:10.1016/s1097-2765(00)80426-8.
114. Singer, B.B.; Scheffrahn, I.; Obrink, B. The tumor growth-inhibiting cell adhesion molecule CEACAM1 (C-CAM) is differently expressed in proliferating and quiescent epithelial cells and regulates cell proliferation. *Cancer Res* **2000**, *60*, 1236-1244.
115. Calinescu, A.; Turcu, G.; Nedelcu, R.I.; Brinzea, A.; Hodorogea, A.; Antohe, M.; Diaconu, C.; Bleotu, C.; Pirici, D.; Jilaveanu, L.B., et al. On the Dual Role of Carcinoembryonic Antigen-Related Cell Adhesion Molecule 1 (CEACAM1) in Human Malignancies. *J Immunol Res* **2018**, *2018*, 7169081, doi:10.1155/2018/7169081.
116. Banerjee, H.; Nieves-Rosado, H.; Kulkarni, A.; Murter, B.; McGrath, K.V.; Chandran, U.R.; Chang, A.; Szymczak-Workman, A.L.; Vujanovic, L.; Delgoffe, G.M., et al. Expression of Tim-3 drives phenotypic and functional changes in Treg cells in secondary lymphoid organs and the tumor microenvironment. *Cell Rep* **2021**, *36*, 109699, doi:10.1016/j.celrep.2021.109699.

117. Schwartz, J.A.; Clayton, K.L.; Mujib, S.; Zhang, H.; Rahman, A.K.; Liu, J.; Yue, F.Y.; Benko, E.; Kovacs, C.; Ostrowski, M.A. Tim-3 is a Marker of Plasmacytoid Dendritic Cell Dysfunction during HIV Infection and Is Associated with the Recruitment of IRF7 and p85 into Lysosomes and with the Submembrane Displacement of TLR9. *J Immunol* **2017**, *198*, 3181-3194, doi:10.4049/jimmunol.1601298.
118. Dixon, K.O.; Tabaka, M.; Schramm, M.A.; Xiao, S.; Tang, R.; Dionne, D.; Anderson, A.C.; Rozenblatt-Rosen, O.; Regev, A.; Kuchroo, V.K. TIM-3 restrains anti-tumour immunity by regulating inflammasome activation. *Nature* **2021**, *595*, 101-106, doi:10.1038/s41586-021-03626-9.
119. Mengshol, J.A.; Golden-Mason, L.; Arikawa, T.; Smith, M.; Niki, T.; McWilliams, R.; Randall, J.A.; McMahan, R.; Zimmerman, M.A.; Rangachari, M., et al. A crucial role for Kupffer cell-derived galectin-9 in regulation of T cell immunity in hepatitis C infection. *PLoS One* **2010**, *5*, e9504, doi:10.1371/journal.pone.0009504.
120. Imaizumi, T.; Kumagai, M.; Sasaki, N.; Kurotaki, H.; Mori, F.; Seki, M.; Nishi, N.; Fujimoto, K.; Tanji, K.; Shibata, T., et al. Interferon-gamma stimulates the expression of galectin-9 in cultured human endothelial cells. *J Leukoc Biol* **2002**, *72*, 486-491.
121. Asakura, H.; Kashio, Y.; Nakamura, K.; Seki, M.; Dai, S.; Shirato, Y.; Abedin, M.J.; Yoshida, N.; Nishi, N.; Imaizumi, T., et al. Selective eosinophil adhesion to fibroblast via IFN-gamma-induced galectin-9. *J Immunol* **2002**, *169*, 5912-5918, doi:10.4049/jimmunol.169.10.5912.
122. Rodrigues Mantuano, N.; Natoli, M.; Zippelius, A.; Läubli, H. Tumor-associated carbohydrates and immunomodulatory lectins as targets for cancer immunotherapy. *J Immunother Cancer* **2020**, *8*, doi:10.1136/jitc-2020-001222.
123. Xue, J.; Suarez, J.S.; Minaai, M.; Li, S.; Gaudino, G.; Pass, H.I.; Carbone, M.; Yang, H. HMGB1 as a therapeutic target in disease. *J Cell Physiol* **2021**, *236*, 3406-3419, doi:10.1002/jcp.30125.
124. Glassman, F.Y.; Dingman, R.; Yau, H.C.; Balu-Iyer, S.V. Biological Function and Immunotherapy Utilizing Phosphatidylserine-based Nanoparticles. *Immunol Invest* **2020**, *49*, 858-874, doi:10.1080/08820139.2020.1738456.
125. Wang, W.; Shi, Q.; Dou, S.; Li, G.; Shi, X.; Jiang, X.; Wang, Z.; Yu, D.; Chen, G.; Wang, R., et al. Negative regulation of Nod-like receptor protein 3 inflammasome activation by T cell Ig mucin-3 protects against peritonitis. *Immunology* **2018**, *153*, 71-83, doi:10.1111/imm.12812.
126. Huang, Y.H.; Zhu, C.; Kondo, Y.; Anderson, A.C.; Gandhi, A.; Russell, A.; Dougan, S.K.; Petersen, B.S.; Melum, E.; Pertel, T., et al. CEACAM1 regulates TIM-3-mediated tolerance and exhaustion. *Nature* **2015**, *517*, 386-390, doi:10.1038/nature13848.
127. Nagahara, K.; Arikawa, T.; Oomizu, S.; Kontani, K.; Nobumoto, A.; Tateno, H.; Watanabe, K.; Niki, T.; Katoh, S.; Miyake, M., et al. Galectin-9 increases Tim-3+ dendritic cells and CD8+ T cells and enhances antitumor immunity via galectin-9-Tim-3 interactions. *J Immunol* **2008**, *181*, 7660-7669, doi:10.4049/jimmunol.181.11.7660.
128. Gleason, M.K.; Lenvik, T.R.; McCullar, V.; Felices, M.; O'Brien, M.S.; Cooley, S.A.; Verneris, M.R.; Cichocki, F.; Holman, C.J.; Panoskaltsis-Mortari, A., et al. Tim-3 is an inducible human natural killer cell receptor that enhances interferon gamma production in response to galectin-9. *Blood* **2012**, *119*, 3064-3072, doi:10.1182/blood-2011-06-360321.
129. Ndhlovu, L.C.; Lopez-Vergès, S.; Barbour, J.D.; Jones, R.B.; Jha, A.R.; Long, B.R.; Schoeffler, E.C.; Fujita, T.; Nixon, D.F.; Lanier, L.L. Tim-3 marks human natural killer cell maturation

and suppresses cell-mediated cytotoxicity. *Blood* **2012**, *119*, 3734-3743, doi:10.1182/blood-2011-11-392951.

130. Kadowaki, T.; Morishita, A.; Niki, T.; Hara, J.; Sato, M.; Tani, J.; Miyoshi, H.; Yoneyama, H.; Masaki, T.; Hattori, T., et al. Galectin-9 prolongs the survival of septic mice by expanding Tim-3-expressing natural killer T cells and PDCA-1+ CD11c+ macrophages. *Crit Care* **2013**, *17*, R284, doi:10.1186/cc13147.
131. Chiba, S.; Baghdadi, M.; Akiba, H.; Yoshiyama, H.; Kinoshita, I.; Dosaka-Akita, H.; Fujioka, Y.; Ohba, Y.; Gorman, J.V.; Colgan, J.D., et al. Tumor-infiltrating DCs suppress nucleic acid-mediated innate immune responses through interactions between the receptor TIM-3 and the alarmin HMGB1. *Nat Immunol* **2012**, *13*, 832-842, doi:10.1038/ni.2376.
132. Smith, C.M.; Li, A.; Krishnamurthy, N.; Lemmon, M.A. Phosphatidylserine binding directly regulates TIM-3 function. *Biochem J* **2021**, *478*, 3331-3349, doi:10.1042/bcj20210425.
133. ElTanbouly, M.A.; Schaafsma, E.; Noelle, R.J.; Lines, J.L. VISTA: Coming of age as a multi-lineage immune checkpoint. *Clin Exp Immunol* **2020**, *200*, 120-130, doi:10.1111/cei.13415.
134. Wang, L.; Rubinstein, R.; Lines, J.L.; Wasiuk, A.; Ahonen, C.; Guo, Y.; Lu, L.F.; Gondek, D.; Wang, Y.; Fava, R.A., et al. VISTA, a novel mouse Ig superfamily ligand that negatively regulates T cell responses. *J Exp Med* **2011**, *208*, 577-592, doi:10.1084/jem.20100619.
135. Wang, L.; Jia, B.; Claxton, D.F.; Ehmann, W.C.; Rybka, W.B.; Mineishi, S.; Naik, S.; Khawaja, M.R.; Sivik, J.; Han, J., et al. VISTA is highly expressed on MDSCs and mediates an inhibition of T cell response in patients with AML. *Oncoimmunology* **2018**, *7*, e1469594, doi:10.1080/2162402x.2018.1469594.
136. Deng, J.; Li, J.; Sarde, A.; Lines, J.L.; Lee, Y.C.; Qian, D.C.; Pechenick, D.A.; Manivanh, R.; Le Mercier, I.; Lowrey, C.H., et al. Hypoxia-Induced VISTA Promotes the Suppressive Function of Myeloid-Derived Suppressor Cells in the Tumor Microenvironment. *Cancer Immunol Res* **2019**, *7*, 1079-1090, doi:10.1158/2326-6066.Cir-18-0507.
137. Shahbaz, S.; Bozorgmehr, N.; Koleva, P.; Namdar, A.; Jovel, J.; Fava, R.A.; Elahi, S. CD71+VISTA+ erythroid cells promote the development and function of regulatory T cells through TGF-β. *PLoS Biol* **2018**, *16*, e2006649, doi:10.1371/journal.pbio.2006649.
138. Dunsmore, G.; Koleva, P.; Ghobakhloo, N.; Sutton, R.; Ambrosio, L.; Meng, X.; Hotte, N.; Nguyen, V.; Madsen, K.L.; Dieleman, L.A., et al. Lower Abundance and Impaired Function of CD71+ Erythroid Cells in Inflammatory Bowel Disease Patients During Pregnancy. *J Crohns Colitis* **2019**, *13*, 230-244, doi:10.1093/ecco-jcc/jjy147.
139. Yasinska, I.M.; Meyer, N.H.; Schlichtner, S.; Hussain, R.; Siligardi, G.; Casely-Hayford, M.; Fiedler, W.; Wellbrock, J.; Desmet, C.; Calzolai, L., et al. Ligand-Receptor Interactions of Galectin-9 and VISTA Suppress Human T Lymphocyte Cytotoxic Activity. *Front Immunol* **2020**, *11*, 580557, doi:10.3389/fimmu.2020.580557.
140. Johnston, R.J.; Su, L.J.; Pinckney, J.; Critton, D.; Boyer, E.; Krishnakumar, A.; Corbett, M.; Rankin, A.L.; Dibella, R.; Campbell, L., et al. VISTA is an acidic pH-selective ligand for PSGL-1. *Nature* **2019**, *574*, 565-570, doi:10.1038/s41586-019-1674-5.
141. Wang, J.; Wu, G.; Manick, B.; Hernandez, V.; Renelt, M.; Erickson, C.; Guan, J.; Singh, R.; Rollins, S.; Solorz, A., et al. VSIG-3 as a ligand of VISTA inhibits human T-cell function. *Immunology* **2019**, *156*, 74-85, doi:10.1111/imm.13001.
142. Vachino, G.; Chang, X.J.; Veldman, G.M.; Kumar, R.; Sako, D.; Fouser, L.A.; Berndt, M.C.; Cumming, D.A. P-selectin glycoprotein ligand-1 is the major counter-receptor for P-selectin

on stimulated T cells and is widely distributed in non-functional form on many lymphocytic cells. *J Biol Chem* **1995**, *270*, 21966-21974, doi:10.1074/jbc.270.37.21966.

143. Moore, K.L.; Eaton, S.F.; Lyons, D.E.; Lichenstein, H.S.; Cummings, R.D.; McEver, R.P. The P-selectin glycoprotein ligand from human neutrophils displays sialylated, fucosylated, O-linked poly-N-acetyllactosamine. *J Biol Chem* **1994**, *269*, 23318-23327.
144. André, P.; Spertini, O.; Guia, S.; Rihet, P.; Dignat-George, F.; Brailly, H.; Sampol, J.; Anderson, P.J.; Vivier, E. Modification of P-selectin glycoprotein ligand-1 with a natural killer cell-restricted sulfated lactosamine creates an alternate ligand for L-selectin. *Proc Natl Acad Sci U S A* **2000**, *97*, 3400-3405, doi:10.1073/pnas.040569797.
145. Wimazal, F.; Ghannadan, M.; Müller, M.R.; End, A.; Willheim, M.; Meidlinger, P.; Schernthaner, G.H.; Jordan, J.H.; Hagen, W.; Agis, H., et al. Expression of homing receptors and related molecules on human mast cells and basophils: a comparative analysis using multi-color flow cytometry and toluidine blue/immunofluorescence staining techniques. *Tissue Antigens* **1999**, *54*, 499-507, doi:10.1034/j.1399-0039.1999.540507.x.
146. Kim, H.; Takegahara, N.; Walsh, M.C.; Middleton, S.A.; Yu, J.; Shirakawa, J.; Ueda, J.; Fujihara, Y.; Ikawa, M.; Ishii, M., et al. IgSF11 regulates osteoclast differentiation through association with the scaffold protein PSD-95. *Bone Res* **2020**, *8*, 5, doi:10.1038/s41413-019-0080-9.
147. Watanabe, T.; Suda, T.; Tsunoda, T.; Uchida, N.; Ura, K.; Kato, T.; Hasegawa, S.; Satoh, S.; Ohgi, S.; Tahara, H., et al. Identification of immunoglobulin superfamily 11 (IGSF11) as a novel target for cancer immunotherapy of gastrointestinal and hepatocellular carcinomas. *Cancer Sci* **2005**, *96*, 498-506, doi:10.1111/j.1349-7006.2005.00073.x.
148. Suzu, S.; Hayashi, Y.; Harumi, T.; Nomaguchi, K.; Yamada, M.; Hayasawa, H.; Motoyoshi, K. Molecular cloning of a novel immunoglobulin superfamily gene preferentially expressed by brain and testis. *Biochem Biophys Res Commun* **2002**, *296*, 1215-1221, doi:10.1016/s0006-291x(02)02025-9.
149. Wakayama, T.; Ohashi, K.; Mizuno, K.; Iseki, S. Cloning and characterization of a novel mouse immunoglobulin superfamily gene expressed in early spermatogenic cells. *Mol Reprod Dev* **2001**, *60*, 158-164, doi:10.1002/mrd.1072.
150. ElTanbouly, M.A.; Zhao, Y.; Nowak, E.; Li, J.; Schaafsma, E.; Le Mercier, I.; Ceeraz, S.; Lines, J.L.; Peng, C.; Carriere, C., et al. VISTA is a checkpoint regulator for naïve T cell quiescence and peripheral tolerance. *Science* **2020**, *367*, doi:10.1126/science.aay0524.
151. Broughton, T.W.K.; ElTanbouly, M.A.; Schaafsma, E.; Deng, J.; Sarde, A.; Croteau, W.; Li, J.; Nowak, E.C.; Mabaera, R.; Smits, N.C., et al. Defining the Signature of VISTA on Myeloid Cell Chemokine Responsiveness. *Front Immunol* **2019**, *10*, 2641, doi:10.3389/fimmu.2019.02641.
152. Rogers, B.M.; Smith, L.; Dezso, Z.; Shi, X.; DiGiammarino, E.; Nguyen, D.; Sethuraman, S.; Zheng, P.; Choi, D.; Zhang, D., et al. VISTA is an activating receptor in human monocytes. *J Exp Med* **2021**, *218*, doi:10.1084/jem.20201601.
